# Death of a Centenarian

**Published:** 1829

**Authors:** 


42. *Death of a Centenarian.*—The journal just quoted, has announced the death of the venerable Edward Augustus

Holyoke, M. D. & L. L. D. aged a hundred years. We have given some account of the honors which the members of the profession in Boston and Salem did themselves the *honor*, a few months since, to pay to this—the oldest physician of the new world. We have made arrangements to obtain an original sketch of the life and character of the deceased, for insertion in a future number.

